# Pulmonary vein capture is a predictor for long-term success of stand-alone pulmonary vein isolation with cryoballoon ablation in patients with persistent atrial fibrillation

**DOI:** 10.3389/fcvm.2023.1150378

**Published:** 2024-02-12

**Authors:** Alexey Babak, Christine Bienvenue Kauffman, Cynthia Lynady, Reginald McClellan, Kalpathi Venkatachalam, Fred Kusumoto

**Affiliations:** ^1^ Department of Cardiovascular Medicine, Mayo Clinic Florida, Jacksonville, FL, United States; ^2^ School of Medicine, Emory University, Atlanta, GA, United States

**Keywords:** atrial fibrillation, cryoballoon, pulmonary vein, persistent atrial fibrillation, ablation, electrophysiology

## Abstract

**Background:**

The mechanisms of AF development and progression are still not completely understood. Despite the relative efficacy of ablation, the risk of AF recurrence is substantial, particularly in patients with persistent AF (perAF). At present we do not have any reliable intra-procedural electrophysiologic predictors of long-term success of AF ablation other than pulmonary vein isolation. We evaluated selected intraprocedural pulmonary vein characteristics that may be helpful in future guidance of persistent AF ablation.

**Methods:**

390 consecutive procedures using cryoballoon for initial AF ablation were divided by clinical presentation (paroxysmal or persistent AF), and by pulmonary vein (PV) response to pacing after completion of ablation (discrete electrogram elicited with pacing—“PV capture” or not—“Control”). Patients were followed (median 20 months) for recurrent atrial arrhythmias as the primary end point of the study.

**Results:**

PV capture was identified in 20.3% and 17.1% and patients with paroxysmal and persistent AF respectively (ns). In patients with persistent AF presence of PV capture was associated with significantly better outcomes compared to patients without PV capture (*p* < 0.001). In the group “persistent AF and PV capture”, an initial strategy of PV isolation and reisolation of the PVs (without additional lesions) for patients with recurrent atrial arrhythmias resulted in 20/23 (87%) patients in sinus rhythm off antiarrhythmic medications at study completion. In patients with paroxysmal AF, PV capture was not associated with outcome benefits. Speciﬁc electrophysiologic characteristics of PV (PV capture cycle length: PVCCL) did not have an impact on AF recurrence, although 25% shortening of PVCCL was observed after 60 s periods of pacing at short cycle lengths. No background demographic patient characteristic differences were identiﬁed between patients with vs. without PV capture.

**Conclusion:**

The presence of PV capture was associated with better outcomes in patients with persistent AF. PV capture may identify those patients with persistent AF in whom cryoballoon PV isolation alone is sufficient as an initial ablation procedure and as the primary ablation strategy for recurrent AF.

## Introduction

Randomized controlled trials in patients with atrial ﬁbrillation have demonstrated that catheter ablation is superior to pharmacological therapy with reduced likelihood of recurrent AF and improved quality of life, and may confer a survival beneﬁt in patients with accompanying congestive heart failure (1–4). Mechanisms underlying AF are complex and remain incompletely understood (1, 2). Despite relative efficacy of ablation, the risk of AF recurrence is still substantial, particularly in patients with persistent AF (1). Atrial substrate modiﬁcation in addition to pulmonary vein isolation (PVI) has historically been thought to be necessary to achieve satisfactory success rates in patients with persistent atrial fibrillation (1). However, several randomized trials demonstrated that adjunctive RF ablation strategies did not result in higher freedom from arrhythmia than a PVI-only strategy but were associated with higher ﬂuoroscopy and procedure times (4, 5). Recently, several studies have suggested that cryoballoon ablation of the PVs may be moderately effective for the initial treatment of patients with persistent AF (6–8). The presence of exit block from the pulmonary veins has been used as the traditional endpoint for effective pulmonary vein ablation (1). Evidence of exit block may be difficult to confirm and often its presence is implied by the presence of entrance block [GL (9)]. While successful production of exit block during cryoballoon ablation has been reported, we evaluated whether electrophysiologic properties of pulmonary veins (PV capture and PV cycle length) after cryoballoon ablation could identify patients with persistent atrial ﬁbrillation might be successfully treated with strategies directed solely at PVI both at the index procedure and any necessary subsequent procedures (10).

## Methods

### Patient population and follow-up

Three hundred ninety consecutive initial PVI procedures using cryoballoon for symptomatic drug-refractory AF were retrospectively evaluated. Patients with self-limited episodes shorter than 7 days duration and no history of prior cardioversion (done for AF episodes less than 48 h duration) were deﬁned as having paroxysmal AF (PAF), patients with AF who did not satisfy these criteria were placed to “persistent AF” group. Patients with a past medical history of atypical atrial ﬂutter or prior ablation for atrial ﬁbrillation were not included in the study. Antiarrhythmic drugs were stopped at least five half-lives prior to the ablation procedure (amiodarone stopped ≥1 month prior). Post-procedure follow-up was performed at a routine cardiology clinic visits at 1 month, 6 months, 12 months, and then annually or earlier as needed according to symptoms, including emergency department visits. All patients underwent ECG monitoring for 7–30 days with duration based on provider preference. Outcome analysis was started 3 months (“blanking period”) after the index ablation procedure. Antiarrhythmic medication was used based on the health provider's judgment during the first 3 months but was stopped after the blanking period.

The primary clinical outcome was recurrence of atrial arrhythmias detected by rhythm monitoring or symptoms reported by a patient at a follow-up visit with the need for additional therapy (cardioversion, re-ablation, drug therapy) or change to a rate control strategy. There were no patients lost to follow-up. Follow-up providers were blinded to electrophysiologic details of the ablation procedure. For patients who underwent a repeat ablation a second blanking period was not instituted.

### Ablation procedure

Patients underwent ablation using a 28 mm cryoballoon (Medtronic Inc., Minneapolis, Minnesota). During procedures, patients were in deep analgosedation. Patients who presented in AF were electrically cardioverted to sinus rhythm at the start of the procedure. If patient rhythm could not be converted to sinus rhythm, the ablation procedure was started with the patient in AF, with additional cardioversion delivered as needed after defined milestones such as pulmonary vein encircling. Temperatures lower than −45°C were always sought with ablation of 240 s, shorter time (180 s) if lower temperatures were achieved (−50°C). Ablation stopped if temperature achieved −55°C. Diaphragmatic pacing techniques were used for identifying phrenic nerve injury during ablation of the right-sided pulmonary veins. Additional radiofrequency ablation of the cavotricuspid isthmus with confirmation of bidirectional block was performed for patients with previously clinically documented typical atrial ﬂutter or if typical atrial ﬂutter was induced or identiﬁed during the index procedure. Patients with inducible atypical atrial ﬂutter underwent cardioversion or atrial pacing to resolve the atypical atrial flutter without additional mapping or ablation.

### Evaluation of electrophysiologic properties of the PVs

**Figure 1:**
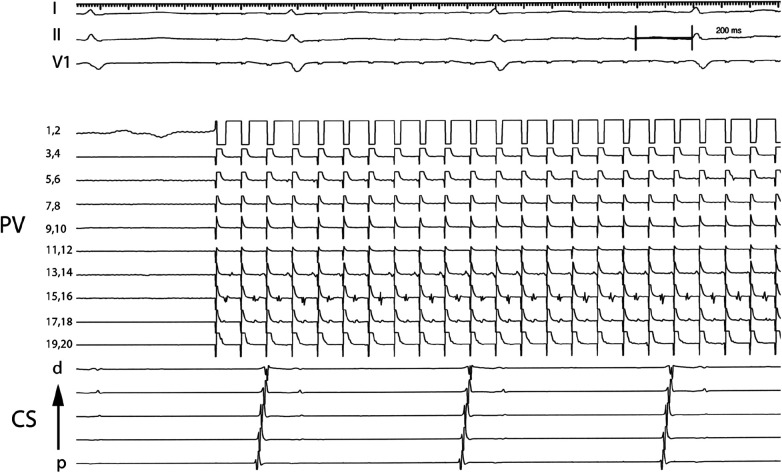
Electrograms demonstrating PV capture dissociated from atrial tissue. Pacing from electrode pair 1,2 at a cycle length of 100 ms results in 1:1 capture in PV tissue recorded by electrode pairs 17,18 and 15,16 and intermittent block in PV tissue recorded by electrode pairs 13,14 and 5,6. PV: Pulmonary vein; CS: Coronary sinus; p: proximal; d: distal.

After PV isolation a multipolar Lasso catheter (Johnson & Johnson Medical, New Brunswick, New Jersey) was placed in each PV, and all aspects of the PV were explored, and the presence of spontaneous PV activity was assessed. The lasso catheter allowed exploration of larger territory at the pulmonary vein os to account for PV anatomy variation, could be manipulated within the pulmonary vein to ensure tissue contact to all aspects of the pulmonary vein, and was less expensive in our experience since it can be reused. Since the lasso catheter was used for longer periods in the left atrium, guiding sheaths were flushed in the right atrium to decrease the potential risk of neurologic events. The PV was paced at multiple sites within the PV, beginning at a cycle length (CL) of 600 ms. PV capture was deﬁned as a repetitive discrete EGM response within PV for more than 10 extrastimulus (10 mA) consecutively (Figure 1). The pacing cycle length was progressively decreased and electrogram behavior within the PV was recorded. When 2:1 PV capture was observed, the cycle length was increased to the point where 1:1 capture was reachieved and deﬁned as the initial PV capture cycle length (PVCCL). After continuous pacing at a higher cycle length for 60 s, the pacing cycle length was then again progressively shortened and in most cases, 1:1 capture was observed at shorter cycle lengths that were previously associated with 2:1 block. The process was continued until no additional decrease in pacing cycle length with 1:1 capture was observed. The ﬁnal PVCCL was deﬁned as the minimum pacing cycle length in which 1:1 capture was observed. The *Δ*PVCCL was the difference between the initial PVCCL and ﬁnal PVCCL. The decision on whether to use the adenosine to demonstrate “dormant” forms of PV-to-atrial conduction was left to the operator (11, 12).

### Data management and statistical analysis

The measured values were checked for distribution type by the Shapiro–Wilk test. Continuous variables with non-normal distribution are represented as median [25th percentile, 75thpercentile], or as mean [ ± standard deviation]. Categorical variables were tested using Chi- square test. Arrhythmia recurrence was evaluated by the Kaplan–Meier method, differences in the freedom from arrhythmia were compared using the log-rank test. Statistical analysis was performed using SPSS version 22.0 (IBM, Armonk, New York, USA) and two-tailed *p*-values <0.05 were deemed to be statistically signiﬁcant. The authors had full access to and take full responsibility for the integrity of the data. All the authors have read and agree to the manuscript as written.

## Results

### Baseline characteristics

For purpose of our study patients undergoing AF ablation were divided into 4 groups depending on the type of AF (“persistent” vs. “paroxysmal”) and PV capture—whether discrete PV electrogram was identiﬁed during PV pacing or not (“PV capture” vs. “Control” group). Figure 1 shows an example of PV capture.

The characteristics of the overall population evaluated were as follows: age 67 [60.0; 72.0] years, follow-up period was 20 [9; 36] months, 72% of patients were males, 53.8% of the patients were taking class III antiarrhythmic and 60.8% were taking beta-blockers before the index procedure, arterial hypertension was comorbidity in 62.3% and DM in 23.8% of cases. There were no signiﬁcant differences between the groups studied besides of utilization of class I antiarrhythmics, which was signiﬁcantly higher in patients with “paroxysmal AF and PV capture” vs. other groups (Tables 1 and 2). Left atrial diameter was not significantly different among patients the different groups.

**Table 1:** Baseline characteristics.

Characteristics	Overall across groups	Group 1	Group 2	Group 3	Group 4	*P*-value
		PV capture (parox)	Control (parox)	PV capture (persist)	Control (persist)	
*N*	390	52	204	23	111	
Gender (M/F),	281/109	38/14	141/63	18/5	84/27	Ns
Age	67[60.0;72.0]	65[58.0;71.0]	67 [61.0;72.0]	67[60.0;73.0]	67[59.0;71.0]	Ns
BMI	30.2 (5.1)	28.7 (5.2)	29.7 (5.5)	30.6 (7.9)	31.9 (7.5)	Ns
CHF, % *(n)*	11.3 (44)	3.8 (2)	11.3 (23)	17.4 (4)	13.5 (15)	Ns
HTN, % *(n)*	62.3 (243)	55.8 (29)	65.2 (133)	52.2 (12)	62.2 (69)	Ns
DM, % *(n)*	23.8 (48)	31.6 (6)	21.0 (21)	37.5 (3)	24.0 (18)	Ns
Class 1 AAM, % *(n)*	10.5 (41)	26.9 (14)^a^	9.8 (20)	8.7 (2)	4.5 (5)	0.001
Class 3 AAM, % *(n)*	53.8 (210)	51.9 (27)	48.0 (98)	73.9 (17)	61.3 (68)	Ns
Diuretic, % *(n)*	21.8 (85)	17.3 (9)	20.1 (41)	21.7 (5)	27.0 (30)	Ns
CCB, % *(n)*	22.6 (88)	36.5 (19)	17.6 (36)	26.1 (6)	24.3 (27)	Ns
BB, % *(n)*	60.8 (237)	59.6 (31)	85.8 (175)	65.2 (15)	59.5 (66)	Ns
ACEI, % *(n)*	42.1 (164)	40.4 (21)	39.7 (81)	52.2 (12)	45.0 (50)	Ns
LAD (mm)	42 ± 5	41 ± 4.5	41 ± 4.3	42 ± 5.1	43 ± 4.9	Ns

BMI, body mass index; CHF, congestive heart failure; HTN, hypertension; DM, diabetes mellitus; AA, antiarrhythmic medication; CCB, calcium channel blocker; BB, beta-blocker; ACEI, angiotensin-converting enzyme inhibitor.

^a^ The predominance of male participants, and significantly older age of female participants (66 [58; 71] vs. 70 [65; 75,5] y.o. respectively) in our study mirrors the overall distribution of AF in population (Table 3). There were no significant cross-gender differences in the distribution of forms of AF and PV properties, as well as in the arrhythmia-free period after ablation. We analyzed the predictive value of baseline characteristics on arrhythmia-free survival by univariate and multivariate Cox proportional hazards regression (Tables 4 and 5). The type of arrhythmia and PV capture were the only predictors of the favorable outcome with both univariate and multivariate analysis. Other indicators evaluated such as age, BMI, usage of medications, and history of diabetes, failed to show significance with multivariate analysis. Group signiﬁcantly differs from each group.

**Table 2:** Pv electrophysiologic characteristics in patients based on arrhythmia type or whether or not recurrent AF was identiﬁed.

	Paroxysmal AF	Persistent AF	Statistics	Recurrence	No recurrence	Statistics
PVCL initial	215 ± 50	218 ± 75	NS	216 ± 66	216 ± 56	NS
PVCL final	160 ± 47	187 ± 54	NS	157 ± 54	177 ± 49	NS
Delta PVCL	56 ± 33	34 ± 24	*p* < 0.05	56 ± 38	43 ± 27	NS

PVCCL, Pulmonary vein capture cycle length, ms.

The predominance of male participants, and significantly older age of female participants (66 [58; 71] vs. 70 [65; 75,5] y.o. respectively) in our study mirrors the overall distribution of AF in population (Table 3). There were no significant cross-gender differences in the distribution of forms of AF and PV properties, as well as in the arrhythmia-free period after ablation.

**Table 3:** Gender distribution of indexes.

	Age	Follow up time	Paroxysmal	Persistent	PV Capture	Control
Male	66 [58;71]	21,5 [10,0; 36,0]	179	102	56	225
Female	70 [65;75,5]	24,0 [10,5; 39,0]	77	32	19	90
Significance	*P* < 0,001	ns	ns		ns	

We analyzed the predictive value of baseline characteristics on arrhythmia-free survival by univariate and multivariate Cox proportional hazards regression (Tables 4 and 5). The type of arrhythmia and PV capture were the only predictors of the favorable outcome with both univariate and multivariate analysis. Other indicators evaluated such as age, BMI, usage of medications, and history of diabetes, failed to show significance with multivariate analysis.

**Table 4:** Predictors of single-procedure arrhythmia-free survival (univariate regression).

Variable	HR	95,0% CI for HR		*P*-value
		Lower	Upper	
Parox/Persist AF	0,534	0,400	0,714	<0,001
PV capture /No capture	0,031	0,014	0,071	<0,001
Gender (F/M)	0,685	0,494	0,951	<0,05
Age	0,855	0,835	0,875	<0,001
Delta PVCL, BMI, Diabetes				ns

**Table 5:** Predictors of single-procedure arrhythmia-free survival (multivariate regression).

Variable	HR	95,0% CI for HR		*P*-value
		Lower	Upper	
Parox/persist AF	0.615	0.463	0.818	0.001
PV capture/no capture	0.577	0.375	0.889	0.013
Gender (F/M)	1.147	0.830	1.587	0.406
Age	1.002	0.986	1.018	0.802
BMI	1.018	0.994	1.041	0.140
Diabetes	0.64	0.416	1.058	0.085

### Outcomes

**Figure 2:**
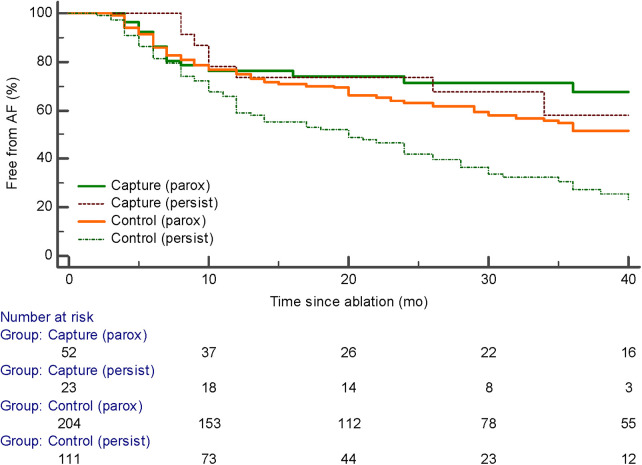
Kaplan–Meier curves of the arrhythmia-free survival after initial ablation procedure.

**Figure 3:**
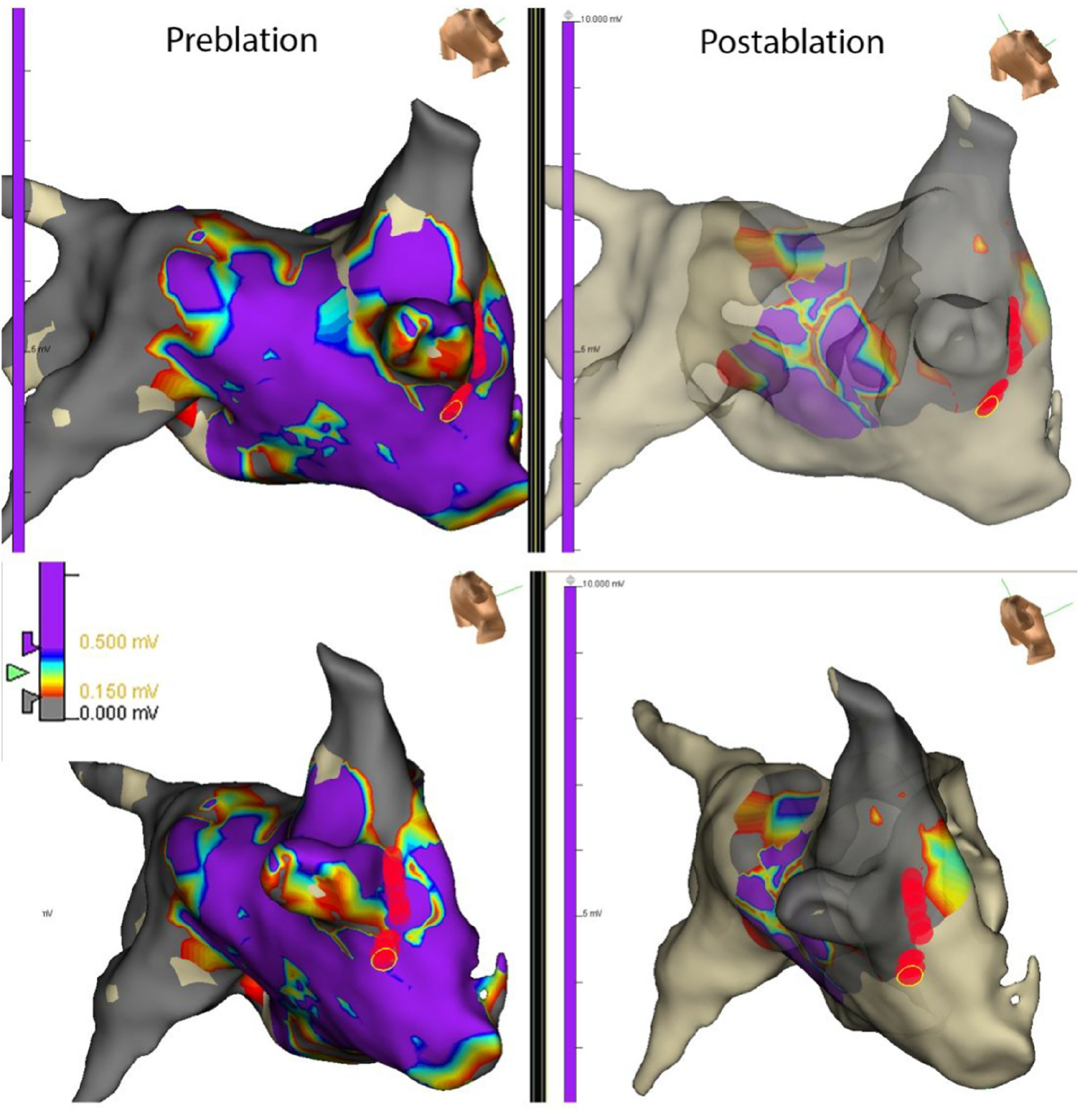
Map from a repeat ablation procedure showing electrograms measured from different regions of the left atrium and PV in a patient with persistent AF and PV capture identiﬁed at the index procedure. Recurrent conduction to the right-sided pulmonary veins was identiﬁed due to recurrent conduction in the septal and inferior regions of the right inferior pulmonary vein. Limited ablation with reisolation of the pulmonary veins (red circles) resulted in the elimination of recurrent AF at follow-up. The apparent changes in voltages noted in the posterior LA in the post-ablation maps are due to undercollection rather than a new scar.

PV capture was present in 52 of 256 (20.3%) of patients with paroxysmal AF and 23 of 134 (17.1%) of patients with persistent AF (ns). The development of recurrent atrial arrhythmias during follow-up was similar in patients with paroxysmal AF with or without PV capture (*p* = ns). The median follow-up was 20 months with no significant difference among the four groups. However, the presence of PV capture in persistent AF identiﬁed a group with favorable ablation outcomes similar to patients with paroxysmal AF. These patients had signiﬁcantly fewer recurrent atrial arrhythmias than patients with persistent AF without PV capture (*p* < 0.001) (Figure 2). Of the 23 patients with persistent AF and pulmonary vein capture, 8 underwent repeat ablation >3 months after the index procedure. At repeat ablation, all patients had recurrent PV conduction and underwent reisolation of the pulmonary veins with no additional atrial lesions (Figure 3). At study completion, 20 of the 23 patients were in sinus rhythm without antiarrhythmic medications. Of the 111 patients with persistent AF and no evidence for pulmonary vein capture, 49 underwent repeat ablation, and 42 of 111 patients were in sinus rhythm without antiarrhythmic medications.

### PV electrophysiologic characteristics

**Figure 4:**
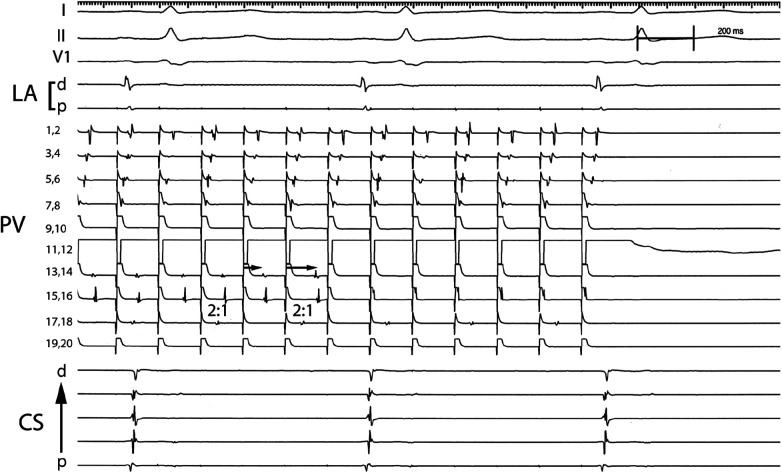
Complex conduction patterns within the PV observed with PV pacing. Pacing from electrode pair 11,12 in the PV results in 1:1 conduction that travels from electrode pairs 7,8 to 1,2 and 2:1 conduction in electrode pair 17,18 (2:1). Progressive delay in conduction is observed that appears to travel from electrode pair 13,14 to 15,16 (arrows) which after block is replaced with rapid 1:1 conduction observed in electrode pair 15,16 but absent activation in electrode pair 13,14. PV: Pulmonary vein; CS: Coronary sinus; p: proximal; d: distal.

For patients with PV capture, the electrophysiologic characteristics of the pulmonary vein tissue were evaluated (Table 2). The minimal PV capture was dynamic. With initial pacing, 2:1 block would be identiﬁed at the cycle length (CL) would vary, and with continued pacing the cycle length inevitably decreased. In several patients the pacing cycle length decreased to values less than 100 ms (Figure 1). In patients with PV capture CL (PVCCL) less than 150 ms, complex conduction patterns were also observed (Figure 4). The electrophysiologic properties of the PV did not differ between patients with paroxysmal or persistent AF and for the initial and ﬁnal PVCCL, although a more signiﬁcant reduction in PVCCL during pacing was noted in patients with paroxysmal AF. The presence of spontaneous dissociated PV potentials was more commonly observed in patients with paroxysmal AF when compared to patients with persistent AF (paroxysmal AF: 9.6% vs. persistent AF 4.3%; *p* < 0.001). PV capture was usually observed in only one PV (in almost all cases, the left superior PV or right superior PV), though 3 patients with paroxysmal AF had PV capture identiﬁed in two PVs. No electrophysiologic characteristics of the pulmonary vein were identiﬁed that had an impact on clinical outcomes for the entire group with PV capture or in patients with paroxysmal or persistent AF.

## Discussion

The present study evaluated whether the characteristics of PV response on pacing during cryoablation are related to long-term AF-freedom. Our results suggest that evidence of PVpotentials (PV capture) after PV isolation is present in 20.3% of patients with paroxysmal AF and 17.1% of patients with persistent AF. The presence of PV capture appears to identify a group of patients with persistent AF in whom PV isolation alone either at the index procedure or at any required follow-up procedure is associated with favorable outcomes. PV capture did not predict the clinical efficacy of PV isolation in paroxysmal AF. In a prior study of patients undergoing catheter ablation using r adiofrequency energy, the presence of dissociated PV potentials was identiﬁed in 19% of patients and their presence did not have an impact on outcomes (13). The majority of patients in this study had paroxysmal atrial fibrillation (74%), and similarly, we found that the presence of PV capture in patients with paroxysmal AF had no impact on the likelihood of recurrent AF. Another related intraprocedural study of 30 patients found that loss of PV capture was identified in 40% of patients acutely after PVI and found that this finding was associated with entrance block (14). Since, the mass of ablated tissue after cryoablation apopears to be larger than pulmonary vein ablation using radiofrequency energy, it may be that local PV capture after cryoablation may identify a subset of patients with persistent atrial fibrillation in whom greater PV muscle mass serves as an important arrhythmia mechanism (15). The difference in the impact of pulmonary vein capture between patients with paroxysmal atrial fibrillation and persistent atrial fibrillation provides indirect additional evidence for the importance of the pulmonary veins in patients with paroxysmal atrial fibrillation regardless of PV sleeve mass, and that patients with pulmonary vein “dependent” atrial fibrillation and larger PV sleeve mass may clinically present with persistent atrial fibrillation. Finally, though probably related, dissociated PV potentials were only observed in a small minority of patients with PV capture after cryoballoon ablation in the current study, and dissociated spontaneous PV potentials and PV capture may have different implications, particularly in the setting of different ablation energy sources (15).

The optimal lesion set for patients with persistent AF has not been established (1). Prior studies using cryoablation have suggested moderate success rates in patients with persistent AF (6–8). It is likely that persistent AF has multiple underlying electrophysiologic mechanisms and identifying the cause in an individual patient will be critical for designing a speciﬁc management or ablation strategy. The current study suggests that the presence of PV capture in patients with persistent AF identiﬁed a subset of patients in whom an initial approach of PV isolation is sufficient not only at initial ablation but also if subsequent ablation is required, even if atypical atrial ﬂutter is induced with atrial pacing protocols. This study along with others emphasize the importance of identifying individual mechanisms that is particularly relevant for patients with persistent atrial fibrillation (16).

Prior studies have shown that heterogeneity of electrophysiologic characteristics of pulmonary vein tissue may be important in the underlying pathophysiology of atrial fibrillation (17, 18). Our study extends these findings and highlights the potential importance of electrophysiologic characteristics in atrial fibrillation. Our results suggest that the PVs have unique electrophysiologic properties such as very short paced PVCCL, in some cases < 100 ms that would develop with the progressive pacing. PVs also displayed rapid rate dependence with signiﬁcant shortening of the PVCCL even after very short periods of tachycardia (60 s) and provides further evidence that “AF begets AF” (19). In addition, complex electrogram patterns suggestive of dynamic development of conduction block within different regions of the pulmonary vein that were dependent on the direction of the depolarization wavefront were identiﬁed similar to what has been described at the PV-left atrial junction and left atrium, but at even shorter cycle lengths (20, 21) However, even though these electrophysiologic characteristics from a mechanistic standpoint would appear to make AF more likely, similar to a previous study that used radiofrequency energy, in the current study the speciﬁc electrophysiologic characteristics of the PV did not have an identiﬁable impact on clinical outcomes in patients with paroxysmal or persistent AF (22). However, in patients with persistent atrial fibrillation, the presence of pulmonary vein capture after cryoballoon ablation may identify a group of patients in whom pulmonary vein isolation alone is sufficient both initially and at any required repeat procedures.

There are several limitations to the current study. The most important limitation is that the results are derived from a single center with relatively small number of participants with persistent AF and PV capture. However, despite the limited number of patients, this design allowed this analysis to be performed, since the ablation strategies for management of patients with paroxysmal AF and persistent AF were consistent and similar. In addition, there are likely many factors that lead to recurrent atrial ﬁbrillation in persistent AF (23). It is notable that a strategy only targeting pulmonary vein isolation at initial study and for repeat ablation resulted in a high likelihood of maintaining sinus rhythm without antiarrhythmic medications in this selected group of patients with persistent AF. Although left atrial diameters were similar among the different study groups, another limitation is the absence of left atrial voltage data, particularly for those patients with persistent atrial fibrillation. A recent meta-analysis suggests that low voltage area-guided substrate modification may have a beneficial effect on outcomes though a randomized controlled trial found that addition ablation at scarred regions identified by MRI and mapping was not associated with improved outcomes (24). However, identifying potential patients with persistent atrial fibrillation in whom pulmonary vein isolation alone could be satisfactory may be a useful strategy to minimize the potential of collateral damage resulting from more extensive ablation in the left atrium.

## Conclusion

Currently, no speciﬁc optimal approach for ablation of persistent AF has been identified, and this likely represents the signiﬁcant heterogeneity in underlying mechanisms in these patients which may partially explain the relative lack of success of trials that have evaluated the use of anatomic ablation strategies. At present, we do not have any reliable intra-procedural electrophysiologic predictors of long-term success of AF ablation in patients with persistent atrial fibrillation. However, the presence of PV capture may identify a group of patients in which PV isolation using a cryoballoon approach is sufficient at the index procedure and if recurrent atrial fibrillation (AF) with evident reconnection of a pulmonary vein, directing efforts toward PV reisolation without additional ablation may be optimal.

## Data Availability

The original contributions presented in the study are included in the article/Supplementary Material, further inquiries can be directed to the corresponding author.
